# The Use of Clonidine in the Treatment of Debilitating Obsessive-Compulsive Behaviors Comorbid With Depression and Post-Traumatic Stress Disorder: A Case Report

**DOI:** 10.7759/cureus.14920

**Published:** 2021-05-09

**Authors:** Ashish Sarangi, Daniel Payberah, Terry McMahon

**Affiliations:** 1 Psychiatry, Texas Tech University Health Sciences Center, Lubbock, USA

**Keywords:** ocd, ocd/ anxiety disorders, depression, depression pathology, ptsd, ptsd diagnosis and treatment

## Abstract

Clonidine is an alpha-2 agonist traditionally used as an antihypertensive, and more recently in the treatment of psychiatric disorders such as attention-deficit hyperactive disorder (ADHD), tic disorders such as Tourette’s syndrome, and post-traumatic stress disorder (PTSD). However, there are scarce data in the literature about the use of clonidine in the treatment of obsessive-compulsive disorder (OCD).

In this report, we present the case of a 16-year-old female suffering from OCD. The first-line treatment with sertraline was not very efficacious in improving her symptoms and even led to worsening of the same. However, subsequent treatment with clonidine resulted in rapid and significant improvement in her condition. We postulate that further research is required to gain more insight into the therapeutic potential of clonidine in OCD patients.

## Introduction

Obsessive-compulsive disorder (OCD) is a chronic and disabling condition classified under the obsessive-compulsive and related disorders (OCRD) group of disorders in the Diagnostic and Statistical Manual of Mental Disorders, Fifth Edition (DSM-5) [1]. Prevailing definitions characterize obsessions as recurring and unwanted thoughts, impulses, or images, while compulsions are defined as recurring physical or mental behaviors resulting from obsessions [1,2-4]. Some studies postulate that the inverse relationship is true - that is, compulsions occur first, and that obsessions are used to rationalize compulsions [1]. Regardless, the majority of patients recognize these unwanted thoughts and behaviors as pathological [1]. Common obsessions and compulsions include rigorous and repetitive cleaning, thoughts regarding the safety of the self and others, aggressive and sexual thoughts, obsession with organization, excessive detail-orientation, excessive jealousy, musical fixation, and avoidance behaviors to evade such obsessions and compulsions [1,2,5].

We discuss the case of a 16-year-old female suffering from OCD. Even though the first-line treatment with sertraline was not very effective for her, subsequent treatment with clonidine led to rapid and significant improvement in her condition.

## Case presentation

The patient was a 16-year-old female who had been referred to our child and adolescent psychiatry clinic for evaluation of depression, anxiety, and non-suicidal self-injurious behaviors (NSSI). The patient’s initial evaluation was conducted in July 2019. At that time, she reported worsening depression and anxiety in the context of difficult interactions with her brother, peers, and romantic partners. She also complained about stress related to academic pressure due to enrollment in AP classes, athletics, and marching band while also working after school. She reported that she had started to engage in self-injurious/cutting behaviors a year ago when she had to see her father in order to place a restraining order. The patient stated that she would cut superficially with a pocketknife on her left forearm and both thighs in order to relieve emotional pain. She also reported a history of trauma caused by witnessing her biological father abuse his girlfriends, which had resulted in nightmares, flashbacks, and avoidance behavior. Both the patient and her parent underwent evaluation under the anxiety rating scale Screen for Child Anxiety Related Disorders (SCARED); both of them scored 42 on the scale. The parent also completed an evaluation based on the Vanderbilt Attention-Deficit Hyperactive Disorder (ADHD) Diagnostic Rating Scale (VADRS), the results of which were negative. The patient subsequently received a diagnosis of severe recurrent major depressive disorder and chronic post-traumatic stress disorder (PTSD). She was started on sertraline titration scheduled 12.5 mg for the first week, 25 mg for the second week, 37.5 mg for the third week, and 50 mg for the fourth week. At subsequent follow-ups in August and December, the patient reported some modest improvements in mood and anxiety. She denied experiencing major side effects from sertraline aside from minor stomach upset, which had resolved spontaneously. However, she reported acute stressors due to difficult interactions with her younger brother, increased academic demands, and dissolution of her peer social group, all of which had resulted in fluctuations in mood and worsening appetite.

The patient’s sertraline dose was increased to 75 mg to address her persistent depressive mood and anxiety. Her weight was noted to be 70 kg, and hence the dose of sertraline was adjusted accordingly. The initial starting dose for adolescents is generally 50 mg/day; however, it was increased to 75 mg/day given our patient's body weight. The patient’s clinical condition deteriorated in January due to the ongoing environmental social stressors. The patient relapsed with NSSI, which necessitated medical evaluation and management in the emergency center. She was referred for inpatient psychiatric hospitalization; however, as there were no available inpatient beds in her locality, the patient was discharged home after establishing a safety plan and remission of suicidal ideations and NSSI. At her father’s suggestion, the patient discontinued sertraline slowly over two weeks due to a lack of efficacy. At the follow-up visit, the patient and her mother made passing remarks about ritualistic behaviors, which included repeated counting, praying multiple times a day, and constantly checking doors and locks. She reported no improvement with the initiation of sertraline based on her subjective experience. The patient denied experiencing obsessions on a daily basis but agreed to take a rating scale evaluation for OCD, the Child Yale-Brown Obsessive Compulsive Scale (CY-BOCS). The results of the rating scale evaluation were significantly symptomatic in terms of both obsessive and compulsive checklists. The total severity was 18, which indicated moderately severe OCD, and established the diagnosis of OCD, and partly explained the persistent nature of her symptomatology and decompensation experienced in January. The sertraline was hence discontinued, and the patient agreed to participate in a trial of long-acting transdermal clonidine at a dose of 0.1 mg/24 hours per week, which had been designed in light of the recent literature describing the links between OCD and hyperarousal of the sympathetic autonomic nervous system. At the next follow-up visit one month later, the patient reported a drastic reduction in obsessions, compulsions, anxiety, and depressive symptoms with clonidine monotherapy. Furthermore, she reported improved academic and social functioning and increased interest in and enjoyment from musical and artistic endeavors. A follow-up CY-BOCS was performed, which resulted in a score of 12, which was well below the initial score of 18 that had indicated moderate severity. The patient elected to continue with clonidine monotherapy in anticipation of further benefits from the full steady-state concentration of clonidine at six weeks.

## Discussion

Diagnosis of OCD according to DSM-5 criteria requires that symptoms lead to significant distress, resulting in at least one hour of time wasted every day, with special emphasis on the impairment of normal daily functions [4,6]. Notably, the patients' own insight into their disorder, as well as the incidence of comorbid tic disorders, play an important role in the diagnosis and prognosis of OCD [4,6]. Furthermore, OCD appears to be very similar to other OCRDs such as Tourette syndrome, body dysmorphic disorder, hoarding disorder, as well as other disorders relating to anxiety, depression, and schizophrenia [4,5]. In fact, one study has reported that over 90% of patients with OCD suffer from a comorbid psychological disorder [6].

Research shows that OCD affects between 1-3% of the general population [1-6] and that OCD is the fourth most common psychological disorder in the United States [7]. Studies have shown inconsistency in the incidence of OCD among different genders, although certain trends have been identified [2]. In childhood, studies show that males exhibit a higher incidence of OCD symptoms, while in adolescence and adulthood, females exhibit a greater frequency of OCD [1,2]. Despite these differences, the strongest socioeconomic risk factor for lifetime OCD is age, with those aged 18-29 years reported to be at the highest risk [1].

Studies have shown that 65.3% of patients who present with OCD for at least 12 months before assessment report severe role impairment on the Sheehan Disability Scale, as well as impairments related to relationships and social functions [1]. Research has shown that OCD correlates with increased mortality as well as reduced quality of life in all domains for patients, and with decreased quality of life in some domains for the caregivers and families of patients [1]. In fact, data suggests that 13% of OCD patients attempt suicide [6].

Despite the prevalence, associated comorbidities, and the debilitating nature of OCD, it still remains an elusive disorder with many reported theories proposed on its etiology. Twin studies and candidate gene studies have indicated a genetic role in the development of OCD, with the modern understanding reflecting a polygenic mechanism [1,5,7]. Neurotransmitter dysfunction is implicated in genetic studies, with a focus on serotonin, glutamate, and catecholamines [1]. Particular emphasis is placed on the cortico-striatal-thalamo-cortical (CSTC) system, with an imbalance between glutamatergic or GABA-ergic systems resulting in OCD-like behaviors [1,4,5]. Some studies have also explored the roles of inflammation and immune dysfunction in the pathophysiology of OCD [1]. Psychologically speaking, research shows dysfunctions in response inhibition, cognitive flexibility, planning, goal-directed behavior, working memory, and error monitoring - all of which present with alterations in neuronal structures and circuits [4,5]. Environmental factors are also implicated in the development of OCD, with an emphasis on birth complications and traumatic life events [1]. Moreover, certain emphasis is placed on the difference between OCD that develops during adolescence and OCD occurring in adulthood. A few studies have shown that OCD can arise from childhood streptococcal infection or basal ganglia neurodegeneration, although more recent research has brought these associations into scrutiny [5]. Early-onset OCD is typically more severe, familial, and associated with more frequent presentation among males [4,5].

Early findings implicated the role of serotonin in the etiology of OCD, with selective serotonin reuptake inhibitors (SSRIs) leading to preferential responses [1]. SSRIs remain the first-line pharmacological treatment for OCD [1,4-7]. Responses to serotonin were improved further with the emergence of selective dopamine D2 receptor antagonists, implicating dopamine in the mechanism of OCD [1,5]. Emphasis on dopaminergic systems in the etiology of other OCRDs such as Tourette’s syndrome, as well as imaging and genetic studies pointing to differences in dopaminergic systems between affected patients and healthy individuals, have led to a great interest in dopamine-focused therapies for OCD [1]. Notably, pharmacological treatments of OCD are usually done in parallel with nonpharmaceutical approaches. Educating patients and their families about the disorder, as well as reinforcing the proper support networks, is crucial for alleviating anxiety and managing OCD [1,4]. Cognitive-behavioral therapy (CBT) combined with exposure and response prevention (ERP) remains the standard psychotherapy treatment for OCD, with studies showing significant efficacy of CBT with ERP for OCD patients [1,2,4,5,7]. Moreover, neuromodulation techniques - including repetitive transcranial magnetic stimulation (rTMS), transcranial direct current stimulation (tDCS), deep transcranial magnetic stimulation (dTMS), vagal nerve stimulation (VNS), deep brain stimulation (DBS), and electroconvulsive therapy (ECT) - provide newer noninvasive and invasive therapy options for patients [1,3-7]. Ultimately, the gold standard for the treatment of OCD is a combination of pharmacological therapy, commonly SSRIs, alongside CBT combined with ERP [2,5-7].

Despite the documented benefits of the established treatments for OCD, the disease remains difficult to manage [1,8], with one study claiming that 30-40% of patients show poor response to modern treatments [7], and another showing that half of the patients with OCD do not fully respond to treatment [1]. Moreover, modern treatment plans for OCD present notable drawbacks. SSRIs are commonly prescribed in higher doses for OCD patients than are for other anxiety and depression disorders, thereby causing adverse side effects in patients, such as gastrointestinal or sexual dysfunction [1]. Treatment with SSRIs reportedly provides only moderate improvements, with about 60% of patients responding to their first medication. Clomipramine, a tricyclic antidepressant, has been shown to be more effective than SSRIs, but the research surrounding this efficacy remains questionable, and clomipramine presents a weaker safety and tolerability profile for patients when compared to SSRIs [1,4,7]. Nonpharmaceutical therapies for OCD are complicated by the difficulty in accessing treatment in a timely manner, difficulty in establishing reliable healthcare provider-patient rapport, embarrassment and reluctance to seek healthcare on the part of the patients, and a general lack of knowledge about the disorder [1,6]. In fact, some studies show that only 14-56% of patients with OCD seek treatment, with a gap of nearly a decade between the development of the disorder and proper diagnosis [4]. Neuromodulation treatment avenues are relatively new, and research shows gaps in efficacy, not to mention concerns with the more invasive procedures they constitute [1,4]. These neuromodulation treatments tend to be reserved for the more severe and resistant cases of OCD [3]. With wide gaps in both the understanding and treatment of OCD, there is a pressing need to explore alternative treatment regimens for patients.

One potential approach to alternative treatments includes looking at psychological disorders by taking into account the shared pathophysiology and comorbidity with OCD, such as in the case with tic disorders [8]. Studies about the comorbidity of OCD and tic disorders have revealed lifetime prevalence rates ranging from 14 to 61.5% of all patients, while studies focused on the prevalence of comorbidity in pediatric patients have shown rates ranging from 37.6 to 42.5% of patients [9]. Moreover, preliminary evidence shows that co-occurring OCD and tic disorders show stronger alterations with respect to dopaminergic and serotoninergic systems than do patients with mutually exclusive OCD or tic disorders when compared with healthy individuals [9]. Although research does show little to no alteration of sympathetic tone in the pathophysiology of OCD [10], such research is sparse and does not evaluate the efficacy of medications altering the adrenergic system in OCD patients. As such, it remains important to explore this treatment option for OCD patients.

Despite advancements in the field of research and some success in creating awareness about OCD, the disease is still considered to be underdiagnosed and undertreated [1,4,9]. According to one study, only 30.9% of severe cases received OCD-specific treatment [11,1]. In light of this reported undertreatment of OCD, gaps in modern treatment efficacies, and an evolving understanding and reclassification of OCD, there is a pressing need to explore more potential avenues for treating OCD.

This report examined the beneficial effects of clonidine on an adolescent female patient with OCD, suggesting therapeutic potential and highlighting the importance of further research on clonidine as a treatment for OCD.

Clonidine has been shown to alter adrenergic activity; it is an alpha-2 adrenergic receptor agonist that decreases sympathetic tone in the cardiovascular system, acting as an antihypertensive medication [12]. Additionally, clonidine exhibits analgesic, sedative, and anxiolytic effects, and it is used in the treatment of psychological disorders such as ADHD, PTSD, and Tourette syndrome [12]. Studies evaluating clonidine as a treatment for OCD are few in number, and further studies are required to more accurately evaluate the effects of clonidine in OCD patients.

While clonidine is FDA-approved in treating hypertension and ADHD, the drug has also been used as a successful off-label treatment for opiate detoxification, PTSD, and tic disorders such as Tourette syndrome (13). Side effects of clonidine include dose-related and time-limited symptoms such as dizziness, drowsiness, depression, orthostasis, bradycardia, dry mouth, and constipation - with rebound hypertension being the primary risk of sudden discontinuation [13]. Clonidine is currently not FDA-approved for the treatment of OCD. 

Research into the treatment of OCD with clonidine is sparse and very limited in scope. Very few studies have attempted to isolate physiological mechanisms of clonidine in treating OCD patients. One study assessed clonidine’s adrenergic impact in OCD physiology, showing that OCD patients had significantly decreased growth hormone response to clonidine when compared to both control patients and when administered growth hormone-releasing hormone [14]. A follow-up study, however, indicated no significant differences in clonidine-induced cortisol response between OCD and control patients, suggesting an indirect action of clonidine in growth hormone response [15]. Two later studies have presented contradicting results to clonidine’s effects on the growth hormone response in OCD patients. One study showed that IV clonidine induced lower growth hormone response in OCD patients when compared to controls [16]. Another clinical trial found significant increases in growth hormone response to clonidine for both OCD and control patients, but without significant difference between the two groups [17]. Moreover, few studies have focused on the straightforward treatment of OCD symptoms using clonidine. One study indicated that treatment with clonidine improved obsessive ideas among the Monroe County School children, although the study had a very small sample size [18]. Another small study indicated that clonidine treatments transiently decreased OCD symptoms in six patients, although they presented with side effects of drowsiness, calmness, and sedation [19]. One clinical trial found that OCD patients showed a significant and immediate reduction in obsessive-compulsive ratings when they are on clonidine instead of placebo, while control patients showed no difference - although it is worth noting that the article discusses statistical concerns about specifically measuring reductions of OCD symptoms in healthy patients [20]. Of note, in this study, clonidine induced greater drowsiness in the OCD group compared to the control patients [20]. Finally, one case study of a 22-year-old woman with OCD since adolescence indicated successful alleviation of OCD symptoms by using clonidine after failed treatments using various other medications and electroconvulsive therapy, with a notable relapse of OCD symptoms after the patient started her clonidine medications irregularly. Ultimately, the modest body of literature on clonidine’s effects on OCD patients presents a limited but preliminary therapeutic potential and warrants further research into the specific use of the drug.

The authors recognize that there are certain limitations to attributing a clear-cut benefit of clonidine for symptomatic OCD in this case. This includes a possible subtherapeutic dose of sertraline (75 mg) in the management of this patient. The dose of sertraline was not further increased as both the patient and her parents were insistent that it did not have any efficacy in resolving symptoms. Also, OCD is known to be a relapsing-remitting disorder, and its natural course may partially explain why the patient's symptoms improved, which might have coincided with the initiation of clonidine. However, the authors do feel that the patient maintained improvement with sustained treatment with clonidine over the course of weeks.

## Conclusions

In light of the limited research into clonidine’s therapeutic use in OCD, our index patient represents another instance of the successful treatment of adolescent OCD using clonidine as a monotherapy even though the associated comorbidities in the patient’s diagnosis at times made the beneficial role of clonidine in the reduction of OCD symptoms questionable. While sertraline, a first-line treatment for OCD, exhibited limited efficacy for our patient whose symptoms still progressed despite raising her dosage, clonidine provided more immediate and effective alleviation of OCD, anxiety, and depressive symptoms. Despite the limitations of this case, which include a possible subtherapeutic dose of sertraline, natural relapsing-remitting course of OCD, and lack of prolonged follow-up, the authors feel that clonidine's role in the treatment of OCD definitely deserves further study. Based on our findings, we postulate that further investigation is warranted to provide more insight into clonidine’s therapeutic potential in OCD.
